# Matrix Metalloproteinase-9 and Haemozoin: Wedding Rings for Human Host and *Plasmodium falciparum* Parasite in Complicated Malaria

**DOI:** 10.1155/2011/628435

**Published:** 2011-05-26

**Authors:** Mauro Prato, Giuliana Giribaldi

**Affiliations:** Dipartimento di Genetica, Biologia e Biochimica, Facoltà di Medicina e Chirurgia, Università di Torino, Via Santena 5 bis, 10126 Torino, Italy

## Abstract

It is generally accepted that the combination of both *Plasmodium falciparum* parasite and human host factors is involved in the pathogenesis of complicated severe malaria, including cerebral malaria (CM). Among parasite products, the malarial pigment haemozoin (HZ) has been shown to impair the functions of mononuclear and endothelial cells. Different CM models were associated with enhanced levels of matrix metalloproteinases (MMPs), a family of proteolytic enzymes able to disrupt subendothelial basement membrane and tight junctions and shed, activate, or inactivate cytokines, chemokines, and other MMPs through cleavage from their precursors. Among MMPs, a good candidate for targeted therapy might be MMP-9, whose mRNA and protein expression enhancement as well as direct proenzyme activation by HZ have been recently investigated in a series of studies by our group and others. In the present paper the role of HZ and MMP-9 in complicated malaria, as well as their interactions, will be discussed.

## 1. Introduction

Among protozoan parasites of the genus *Plasmodium*, *P. falciparum* is the most deadly agent of human malaria, causing a broad spectrum of clinical manifestations ranging from asymptomatic to severe multiorgan disease. Despite recent major efforts by the research community, malaria remains one of the major diseases in poor areas, including Sub-Saharan Africa and South-East Asia. It is associated with several million clinical cases per year and leads annually to over one million deaths [1, 2]. The pathophysiology of severe malaria complications is not well understood. In some cases, including cerebral malaria (CM), renal failure, lung pathology and malaria during pregnancy, it appears associated with cytoadherence and sequestration of *P. falciparum*-parasitized red blood cells (pRBCs) to vascular endothelium, leading to microcirculatory obstruction, tissue hypoxia, and metabolic disturbances [3–5]. In some cases additional leukocyte extravasation has been reported [6, 7].

As described in the following sections, either pRBCs or parasite products such as haemozoin (HZ, malarial pigment), a lipid-enriched ferriprotoporphyrin IX crystal derived from haemoglobin catabolism by the parasite [8], can modulate the functions of mononuclear and endothelial cells and promote the production of proinflammatory molecules and other soluble factors, including matrix metalloproteinases (MMPs). MMPs are a well-known family of proteolytic enzymes able to disrupt subendothelial basement membranes [9, 10], to destroy tight junctions [11], and to shed, activate, or inactivate cytokines, chemokines, and other MMPs through cleavage from their precursors [12–14]. In the last decade, growing evidence on involvement of MMPs in *falciparum* malaria became available: human* postmortem* studies showed enhanced protein levels of MMP-1 in brains of CM patients [15], whereas MMP-8 was found increased in plasma of severe malaria patients [16]; additionally, activation of the human MMP-9 gene by *P. falciparum* has been reported in microarray studies on whole blood from children with malaria [17]. Interestingly, a role for MMPs during malaria is suggested also by evidence from nonhuman models of CM: in the brain of mice infected by *P. Berghei *ANKA, the etiological agent of murine CM, increased MMP-2, MMP-7, and MMP-9 levels, and pro-MMP-9 activation were found [18, 19].

The present paper will explore the effects of HZ on functions of mononuclear and endothelial cells, focusing on regulation of human MMP-9, which at present among the malaria-related MMPs is the most studied and could be a potential target for adjunctive therapy of complicated severe malaria.

## 2. Effects of HZ on Human Mononuclear and Endothelial Cells

HZ is a birefringent crystalline material made of Fe^3+^-Protoporphyrin IX dimers that derives from the degradation of haemoglobin by intraerythrocytic *Plasmodium* [8]. Unpurified HZ contains also unspecifically attached polyunsaturated fatty acids (PUFAs) such as arachidonic and linolenic acids, originating from membranes of *Plasmodium* digestive vacuoles [20]. The presence in unpurified HZ of large quantities of ferric haem with small amounts of free iron makes HZ a generator of oxidative radicals capable of forming lipoperoxides or other breakdown products from PUFAs (quasilipoxygenase activity) [21]. Analysis of the lipid fraction isolated from native HZ showed large amounts of hydroxyeicosatetraenoic acids (HETEs), hydroxyoctadecadienoic acids (HODEs), and the terminal aldehyde 4-hydroxynonenal (4-HNE) [22].

**Figure 1:**
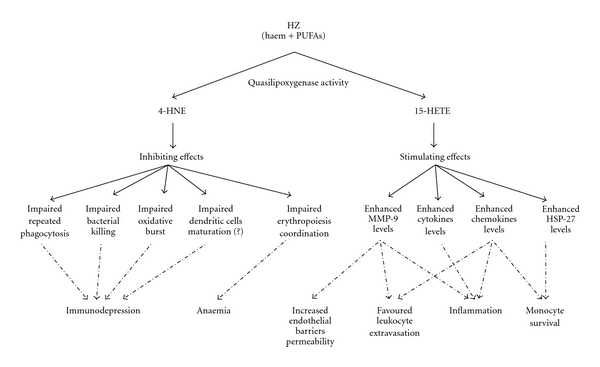
Effects of phagocytosis of HZ on human monocyte functions.

HZ is avidly phagocytosed by human monocytes, and the fatal risk for patients with severe *falciparum* malaria has been reported to be directly proportional to levels of HZ-containing monocytes in peripheral blood [25]. After phagocytosis of HZ, several monocyte functions are dramatically impaired, as shown in Figure 1. The effects of HZ on human monocytes are either inhibiting or stimulating. A detrimental case of functional inhibition led by HZ phagocytosis is represented by immunodepression. As evidenced from *in vitro* studies, HZ-fed human monocytes showed impairment of repeated phagocytosis [26], bacterial killing abilities [27], and oxidative burst [28]. Additionally HZ-laden monocytes did not respond to IFN-gamma stimulation and failed in MHC Class II expression, with following disturbances in antigen presentation [29]. These effects appear to be dependent on the lipid moiety of HZ, and low doses of 4-HNE have been reported to mimic HZ action by inhibiting the IFN-gamma-mediated MHC Class II expression [30]. On the contrary, the inhibitory role of HZ on monocyte differentiation/maturation to dendritic cells is less clear, since similar *in vitro* studies by different authors led to opposite results, showing either inhibition [31] or enhancement [32, 33] of differentiation/maturation to dendritic cells. HZ appears also to be related indirectly to anaemia, as HZ-fed monocytes are not able to coordinate the maturation of erythroid precursors; also in this case a role for 4-HNE in HZ-dependent inhibition of erythropoiesis has been proposed [34]. Regarding the stimulating effects of malarial pigment, HZ-laden monocytes have been shown to overproduce a large series of proinflammatory molecules, including cytokines (TNF, IL-1 beta, and IL-1RA), chemokines (IL-8/CXCL8, ENA-78/CXCL5, GRO*α*, GRO*β*, GRO*γ*, MIP-1*α*, MIP-1*β*, and MCP-1) and the cyto/chemokine-related proteolytic enzyme MMP-9 [23, 35–38]. The lipid moiety of HZ appears to be responsible for these effects, and a major role for 15-HETE has been proposed. Due to the important direct connections to MMP-9, the mechanisms underlying the HZ-triggered pro-inflammatory response will be deeply discussed in a following dedicated section. Curiously, although HZ compromises a large number of monocyte functions, leading to rapid inflammatory response, it has been recently shown that HZ-laden monocytes do not undergo apoptosis. To explain such an apparent dichotomy, a role for heat shock protein-27 (HSP-27), a chemokine-dependent molecule with antiapoptotic properties, has been recently proposed by our group. Indeed, in two models *in vitro* we showed HZ-dependent upregulation of HSP-27 either in immunopurified monocytes from peripheral blood or in the THP-1 monocytic cell line [38, 39].

Anyway, phagocytic cells such as monocytes are not the only human cells which appear to be influenced by HZ. Few data are also available on direct alterations of the human endothelial cell phenotype by HZ. Such a change might be dramatically detrimental during CM, since endothelial cells are a crucial component of the blood-brain barrier (BBB). At present, HZ has been reported to enhance either basal or cytokine-induced levels of adhesion molecules [40, 41] and to inhibit the release of the vasoconstrictor mediator ET-1 [42–44]. Additionally, recent evidence from human microvascular endothelial cells (HMECs) as well as human umbilical vein endothelial cells (HUVECs) showed enhancement or de novo induction of protein expression and activity of several MMPs, including MMP-1, MMP-3, and MMP-9. This topic will be deepened in the following sections.

## 3. Involvement of MMP-9 in Complicated Severe Malaria

MMP-9 is produced by several cells, including mononuclear and endothelial cells. 

Structurally, MMP-9 shares with the other members of the MMP family the following conserved domains: a prodomain, an active domain, a Zn^2+^-binding domain and a carboxyterminal haemopexin domain (which is absent in MMP-7 and MMP-26). In addition MMP-9, as well as the other gelatinase MMP-2, has a gelatin-binding fibronectin domain, composed of three fibronectin repeats, inserted between the active-site domain and the Zn^2+^-binding domain, plus an additional collagen type V domain in a particular hinge region. The Zn^2+^-binding domain, which contains three histidines responsible for the coordination of the catalytic Zn^2+^ ion, forms the active site with the active domain and is required for the enzymatic activity. The fourth ligand of the Zn^2+^ ion is a cysteine of the prodomain. This propeptide can be removed by proteolysis or distorted by substrate binding. The haemopexin domain plays a role in the substrate specificity and is important for the binding of the tissue inhibitors of metalloproteinases (TIMPs). The fibronectin repeats are related to gelatin, laminin, and collagens I/IV binding [9, 10, 45]. 

In the last decade, few evidence of direct involvement of MMP-9 in severe malaria has been reported. Activation of the human MMP-9 gene by *P. falciparum* has been shown in microarray studies on whole blood from children with severe malaria [17], whereas MMP-9 increases in cerebrospinal fluid levels were not apparent, possibly because of the enzyme's tight association with the extracellular matrix [46]. At present no studies of MMP-9 in human *postmortem* brain tissues have been reported, but MMP-9 levels have been shown to be strongly upregulated in C57BL/6 mice brain infected with *P. berghei* ANKA, a murine model of CM sharing similar characteristics with human CM. Elevated MMP-9 levels were selective for the central nervous system, where they were found associated with the vasculature and parenchyma whereas immunohistochemistry showed that higher amounts of MMP-9 were produced by cells of monocytic lineage (CD11b+) [18].

Activation of pro-MMP-9 by removal of the propeptide may occur through several proteolytic mechanisms. It is well known that it can be processed and further activated through a proteolytic cascade involving plasminogen, MMP-3 and MMP-1 [13, 47–49]. Interestingly, an accumulation of urokinase-type plasminogen activator receptor (uPAR) and MMP-1 has been described in brain of patients who died with CM [15, 50]. Furthermore, increased levels of MMP-3 in brain and spleen were observed in the murine model of CM previously cited [18]. Alternative mechanisms for pro-MMP-9 activation involve MMP-2 [51] and MMP-7 [52]. These molecules have not been studied yet in human malaria. However, in CM murine models, MMP-2 mRNA and protein were upregulated in brain [18] and MMP-7 mRNA was increased in liver but not in brain [18], whereas MMP-7 protein was increased in brain [19].

After secretion and activation, MMP-9 activity can still be regulated by degradation or inhibition. MMP-9 is inhibited not specifically by alpha2-macroglobulin and specifically by TIMPs, TIMP-1 showing the highest affinity for MMP-9 [53]. Inhibition of the activated MMP-9 occurs through interaction between the N-terminal domains of TIMP-1 and the active site of the enzyme [54]. The C-terminal part of MMP-9 is also involved, and seems to be responsible for high-affinity interaction with TIMP-1 [55]. Recently, Dietmann et al. measured serum levels of several TIMPs and MMPs in patients with severe malaria [16] and found that elevated TIMP-1 was associated with signs and symptoms of severe malaria; in the same study the authors detected increased levels of MMP-8, but not of MMP-9. MMP-9 is also inhibited by a large number of synthetic compounds [56–58]. Interestingly, antimalarial artemisinin- and artemisinin-derived drugs also showed anti-MMP-9 properties [59–62].

Functionally, MMP-9 is able to process a large pattern of molecules, including matrix proteins, inflammatory factors, surface molecules, and intercellular junctions [12, 14]. Among these substrates, several have been associated to severe malaria.  Table 1 lists the principal MMP-9 substrates which might be relevant for malaria studies. The indicated molecules are either soluble factors (cytokines, chemokines) or membrane-associated proteins (pro-TNF, cell surface proteins, and intercellular junctions). All these molecules appear to be crucial for the development of complications of severe malaria, and their overbalanced regulation is often associated to CM. This is characterized by cytoadherence and sequestering of pRBCs to brain endothelial cells through ICAM-1, followed by an elevated inflammatory response, with high production of TNF and IL-1 beta, as well as several chemokines, which might recruit mononuclear cells and favour monocyte extravasation through the blood-brain barrier (BBB), which appears leaky due to the disruption of tight junctions ZO-1, claudin-5, and occludin [6, 7, 83–90]. Thus, in order to better understand and eventually prevent the mechanisms underlying CM, in the future it will be certainly intriguing to investigate the effects of MMP-9 on these molecules in complicated severe malaria models. Interestingly, the gene induction as well as the direct activation of MMP-9 by HZ has been demonstrated *in vitro* either in mononuclear or on endothelial cells, and an active role for HZ-dependent enhancement of soluble TNF production by human monocytes has already been demonstrated [23], as it will be discussed in the following section.

## 4. Interactions between HZ and MMP-9

The relationship between *falciparum* HZ and human MMP-9, as well as several related molecules, has been investigated in-depth in a series of recent works. In an elegant work Geurts et al. demonstrated that pro-MMP-9 binds directly to the beta-haematin core of HZ through its haemopexin domain, resulting in priming of the activation of the zymogen by other MMPs [91]. In human monocytes HZ has been reported to influence the MMP-9 gene and protein expression, as well as following protein release. Experiments by Real Time RT-PCR have shown that HZ and HZ-containing trophozoites enhanced the mRNA expression of MMP-9 [23]. HZ-dependent MMP-9 gene induction was also confirmed by additional microarray [92] and macroarray [38] studies. As a consequence, pro-MMP-9 protein expression was found to be increased [23]. The degranulation of gelatinase granules with MMP-9, lysozyme, and TIMP-1 was also promoted, as demonstrated by data showing HZ/trophozoite-dependent enhanced release of active MMP-9 [23], lysozyme [93, 94], and TIMP-1 [94] in monocyte cell supernatants. As a result, the total gelatinolytic activity of monocytes was increased by HZ [94] and cells were able to migrate through extracellular matrix [23]. 

The production of several soluble factors related to MMP-9 has also been studied. HZ and trophozoites were shown to stimulate the release of TNF [23] and IL-1 beta [37] by human monocytes. These cytokines, which can modulate the MMP-9 gene expression [95, 96], have been shown to play a crucial role in HZ-dependent enhancement, since the use of anti-TNF and anti-IL-1beta antibodies abrogated the previous HZ effects on pro-MMP-9 expression and active MMP-9 release [23, 37]. Anti-TNF antibodies also blocked the HZ-dependent release of active lysozyme [93]. Interestingly, pro-TNF and pro-IL-1 beta are also two molecular substrates of MMP-9 (see Table 1); as a consequence of cleavage by MMP-9, the soluble TNF is shed from its membrane-bound precursor [63], whereas IL-1 beta is directly activated after removal of the propeptide [64]. The use of a synthetic inhibitor of MMP-9 was shown to abrogate the HZ-dependent enhancement of TNF release by human monocytes [23]. Such an evidence demonstrated that during malaria a pathological autoenhancing loop between MMP-9 and TNF is likely, and the inhibition of MMP-9 could be particularly important in order to avoid detrimental inflammatory effects during complicated malaria, CM above all. On the other hand, the MMP-9 inhibitor did not alter the HZ-enhanced release of IL-1 beta [37], suggesting that in HZ-fed monocytes the production of IL-1 beta precedes the MMP-9 enhancement. This hypothesis was confirmed by recent time-dependent macroarray studies on expression of inflammatory genes, showing that praecox IL-1 beta mRNA expression was already present 2 h after exposure of human monocytes to HZ; therefore, IL-1beta was rapidly endorsed by additional transcription of other inflammatory genes, including MMP-9, TNF, IL-1RA, and several chemokines (IL-8/CXCL8, ENA-78/CXCL5, GRO-alpha/CXCL1, GRObeta, GRO-gamma, MIP-1 alpha, MIP-1 beta, and MCP-1) [38]. Moreover, crossed experiments with anti-TNF and anti-IL-1beta blocking antibodies suggested that the production of IL-1 beta was earlier than TNF production [37].

**Figure 2:**
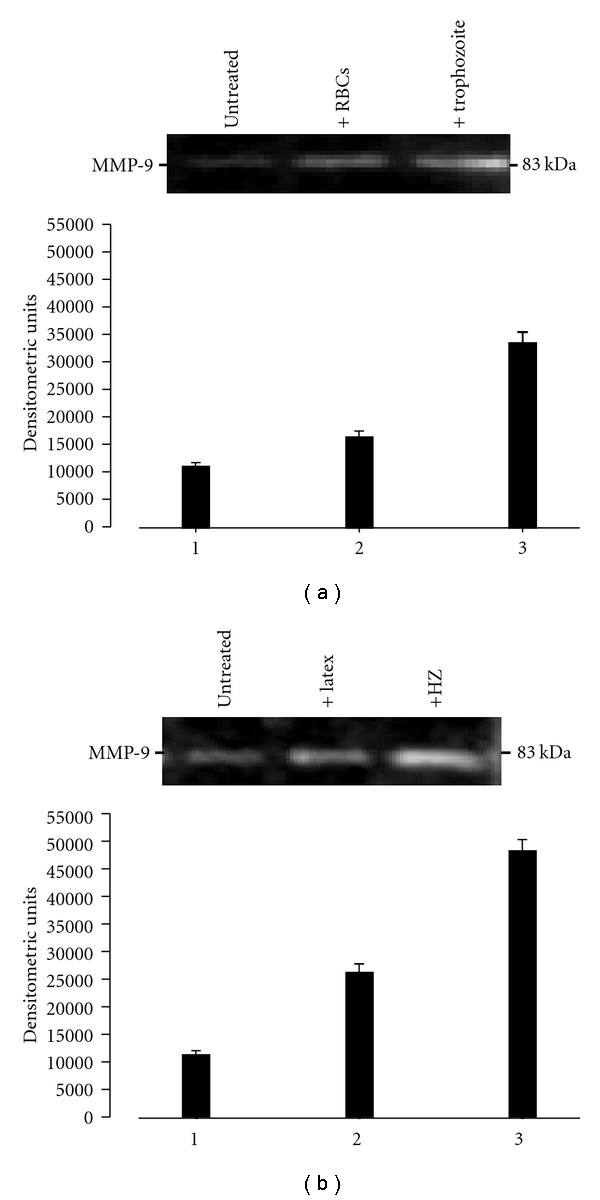
HZ-containing pRBCs (trophozoites) and free HZ enhance MMP-9 levels in HUVECs. Untreated cells (column/lane 1, both panels), cells treated with uninfected RBCs or latex as control stimuli (column/lane 2, (a) and (b) resp.), and cells treated with HZ-containing-trophozoites or HZ (column/lane 3, (a) and (b), resp.) were incubated for 48 h. At the end of the treatment, MMP-9 levels were measured in HUVEC supernatants by SDS-PAGE gelatin zymography and densitometric analysis through a computerized densitometer as previously described [23, 24]. Column data (lower panels) are mean values of arbitrary densitometric units + SEM of three independent experiments; gel data (upper panels) are from one representative experiment; the 83-kDa negative bands in the gel indicate MMP-9 levels. Data were analyzed for significance by Student's *t*-test and all differences were significant ((a) 1 versus 2: *P* < .02; 1 versus 3: *P* < .001; 2 versus 3: *P* < .002. (b) 1 versus 2: *P* < .001; 1 versus 3: *P* < .0001; 2 versus 3: *P* < .001).

Recently, the effects of HZ on MMP-9 regulation have been also studied in endothelial cells. MMP-9 protein expression and release was shown to be dose-dependently induced de novo by HZ in HMECs and to enhance total gelatinolytic activity; as a consequence, cells displayed morphological changes, showing elongated shape and increased ability to form microtubule-like structures through extracellular matrix. In the same work, the enhanced expression of MMP-1 and MMP-3, two enzymes causally connected in the cascade of activation of pro-MMP-9, was also described [97]. Additional data for endothelial cells from large calibre vessels are described in Figure 2, showing that HZ and HZ-containing trophozoites were able to enhance basal levels of activated MMP-9 released in cell supernatants from HUVEC cultures.

Further studies performed either on mononuclear or endothelial cells were aimed to clarify what component (haem or lipid moiety) was responsible for the effects of HZ on MMP-9 and related soluble factors described above. In human monocytes the use of delipidized native or lipid-free synthetic HZ did not reproduce the enhancing effects of HZ on MMP-9 levels [37], gelatinolytic activity [94], lysozyme release [94], and production of TNF [98], IL-1 beta [37], and several chemokines [38], suggesting a major role for the lipid moiety of HZ. Further investigation suggested that a valid candidate responsible for HZ stimulatory effects could be 15-HETE, since micromolar doses of this compound mimicked HZ effects on all previous parameters [37, 38, 94, 98]. A role for the lipid moiety of HZ has been also proposed to explain the HZ effects on HMECs, since delipidized and synthetic HZ did not affect MMP-9, MMP-3, and MMP-1 protein expression [97].

**Figure 3:**
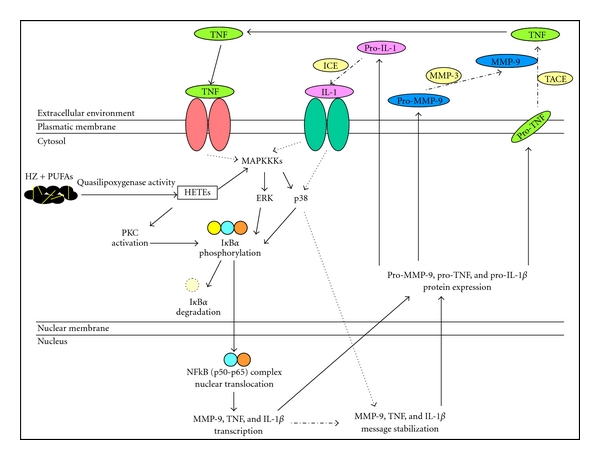
Putative mechanisms through which HZ and 15-HETE might promote NF-kappaB-controlled gene expression of MMP-9 and related molecules.

Finally, a mechanism involving NF-kappaB activation has been recently proposed to explain how HZ and 15-HETE might promote the production of MMP-9 and related molecules in human monocytes. After phagocytosis of HZ and trophozoites, as well as stimulation with 15-HETE, the phosphorylation and degradation of cytosolic IkappaB-alpha protein and the following nuclear translocation of the NF-kappaB complex were promoted either in short-term (2 h after phagocytosis) or long-term (24 h after phagocytosis) studies. These events appeared directly related to HZ-dependent enhancement of MMP-9 release and production of TNF and IL-1 beta, since the use of NF-kappaB inhibitors showing antimalarial properties (quercetin, artemisinin, and parthenolide) abrogated the effects of both HZ and 15-HETE [61]. Additionally, the same inhibitors also abrogated the 15-HETE enhancing effects on total gelatinolytic and lysozyme activity [94]. In another study using a monocytic cell line (THP-1), an extract from a plant with antimalarial properties (*Punica granatum*) inhibited HZ-dependent enhancement of MMP-9 expression and secretion by blocking the NF-kappaB-driven transcription [99]. The previous literature data have shown that HETEs are peroxisome proliferator-activated receptor-gamma ligands inhibiting the production of inflammatory cytokines and suppressing the NF-kappaB system. However, alternative mechanisms which might lead to HETEs-driven NF-kappaB activation have also been documented. A first alternative activation of NF-kappaB is based on HETE-mediated activation of protein kinase C [100–102] which in turn activates IKK [103], the kinase responsible for IkappaB-alpha phosphorylation and degradation [104], and subsequent nuclear translocation of NF-kappaB. Transient activation of protein kinase C by HZ [105] may thus explain the first NF-kappaB activation peak revealed 2 h after HZ phagocytosis. A second alternative activation of NF-kappaB [106] was found to involve MAP kinases, ERK, and p38 cascade. The p38 cascade was also proposed for HETEs-dependent modulation of TNF [107] and MMP-9 [108]. Interestingly, ERK-1/2 phosphorylation by HZ has been documented in a murine system [109]. A third alternative activation of NF-kappaB may result from the direct oxidation and inactivation of IkappaB by lipid hydroperoxides [110].  Figure 3 summarizes the putative mechanisms through which HZ and HETEs might activate the NF-kappaB transcription system and promote the following enhancement of gene induction of MMP-9 and related cytokines.

## 5. Conclusions

Evidence of the involvement of MMPs in complicated severe malaria has been emerging during the last decade, and HZ-dependent induction of MMP-9 expression and activity has been demonstrated in mononuclear or endothelial cells. Since several MMP inhibitors are already available as potential therapeutic tools in other pathologies [55–57], MMPs should be taken in account as potential new targets for an innovative and adjunctive therapy for severe malaria.

## Figures and Tables

**Table 1:** Substrates of MMP-9 that might be critical to complicated severe malaria.

MMP-9 substrate	Functional classification	Effect of MMP-9 proteolytic activity	References
Pro-TNF	Cytokine proform	Shedding	[63]
Pro IL-1beta	Cytokine proform	Activation	[64]
IL-1beta	Cytokine	Degradation	[65]
Pro-TGF-beta	Cytokine proform	Activation	[66]
GRO-alpha/CXCL1	Chemokine	Degradation	[67]
PF-4/CXCL4	Chemokine	Degradation	[67]
ENA-78/CXCL5	Chemokine	Transient potentiation, further inhibition	[68]
GCP-2/CXCL6	Chemokine	No effects	[68]
NAP-2/CXCL7	Chemokine	Degradation	[67]
IL-8/CXCL8	Chemokine	Potentiation	[67]
MIG/CXCL9	Chemokine	Inhibition	[69]
IP-10/CXCL10	Chemokine	Degradation	[69]
SDF-1/CXCL12	Chemokine	Inhibition	[70]
ICAM-1	Cell surface protein	Inhibition	[71]
IL-2R-alpha	Cell surface protein	Inhibition	[72]
Occludin	Intercellular junction	Degradation	[73]
ZO-1	Intercellular junction	Degradation	[74]
ZO-2	Intercellular junction	Degradation (hypothesis)	[75]
Claudin-1	Intercellular junction	Degradation	[76]
Claudin-2	Intercellular junction	Degradation (hypothesis)	[77]
Claudin-4	Intercellular junction	Activation (hypothesis)	[78]
Claudin-5	Intercellular junction	Degradation (to be further investigated)	[79]
Syndecan-1	Structural protein	Shedding	[80]
MBP	Structural protein	Degradation	[81]
Beta-dystroglycan	Structural protein	Degradation	[82]

## Abbreviations

HZ: Haemozoin
MMP: Matrix metalloproteinase
CM: Cerebral malaria
HMEC: Human microvascular endothelial cell
HUVEC: Human umbilical vein endothelial cell
BBB: Blood-brain barrier
MHC: Major histocompatibility complex
PUFA: Polyunsaturated fatty acid
HETE: Hydroxyeicosatetraenoic acid
HODE: Hydroxyoctadecadienoic acid
HNE: Hydroxynonenal
TIMP: Tissue inhibitor of metalloproteinase
TNF: Tumor necrosis factor
IL: Interleukin
ICAM: Intercellular adhesion molecule
uPAR: Urokinase-type plasminogen activator receptor
TGF: Transforming growth factor
ZO: Zonula occludens
MBP: Mannose binding protein
GRO: Growth-regulated oncogene
PF: Platele factor
ENA: Epithelial neutrophil activating peptide
MIP: Macrophage inflammatory protein
MCP: Monocyte chemoattractant protein
GCP: Granulocyte chemotactic protein
NAP: Neutrophil attractant-activation protein
MIG: Monokine induced by interferon-gamma
IP: Interferon-gamma-inducible protein
SDF: Stromal derived factor
ET: Endothelin
HSP: Heat shock protein.
